# Poly[[μ_4_-3,4,8,10,11,13-hexa­hydro-1*H*,6*H*-bis­([1,4]di­thio­cino)[6,7-*b*:6′,7′-*e*]pyrazine]di-μ-iodido-dicopper(I)]: a two-dimensional copper(I) coordination polymer

**DOI:** 10.1107/S2414314620004678

**Published:** 2020-04-07

**Authors:** Tokouré Assoumatine, Helen Stoeckli-Evans

**Affiliations:** a Institute of Chemistry, University of Neuchâtel, Av. de Bellevax 51, CH-2000 Neuchâtel, Switzerland; b Institute of Physics, University of Neuchâtel, rue Emile-Argand 11, CH-2000 Neuchâtel, Switzerland; Vienna University of Technology, Austria

**Keywords:** crystal structure, copper(I) iodide, pyrazine, pyrazine­thio­phane, two-dimensional coordination polymer, supra­molecular framework

## Abstract

The reaction of ligand 3,4,8,10,11,13-hexa­hydro-1*H*,6*H*-bis­([1,4]di­thio­cino)[6,7-*b*:6′,7′-*e*]pyrazine (**L**) with CuI led to the formation of a two-dimensional coordination polymer, incorporating a [Cu_2_I_2_] motif. These units are linked *via* the four S atoms of the ligand to form the title two-dimensional coordination poly­mer, poly[[μ_4_-3,4,8,10,11,13-hexa­hydro-1*H*,6*H*-bis­([1,4]di­thio­cino)[6,7-*b*:6′,7′-*e*]pyrazine]di-μ-iodido-dicopper(I)], [Cu_2_I_2_(C_12_H_16_N_2_S_4_)]_*n*_, (**I**). The asymmetric unit is composed of a ligand mol­ecule, two copper(I) atoms and two I^−^ ions. Both copper(I) atoms are fourfold S_2_I_2_ coordinate with almost regular trigonal-pyramidal environments. In the crystal, the layers, lying parallel to (102), are linked by C—H⋯I hydrogen bonds, forming a supra­molecular framework.

## Abstract 2

The reaction of the pyrazine­thio­phane ligand 3,4,8,10,11,13-hexa­hydro-1*H*,6*H*-bis­([1,4]di­thio­cino)[6,7-*b*:6′,7′-*e*]pyrazine with CuI led to the formation of a two-dimensional copper(I) coordination polymer with Cu^I^ in a trigonal–pyramidal coordination environment defined by two S and two I atoms.

## Graphical abstract

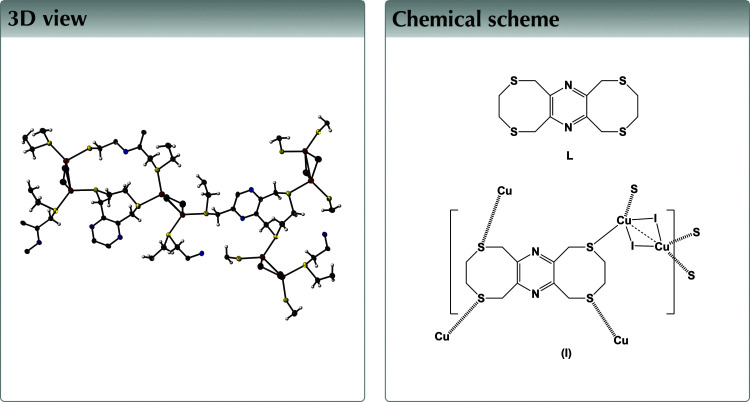

## Structure description

We have recently shown that the reaction of the title pyrazine­thio­phane ligand, 3,4,8,10,11,13-hexa­hydro-1*H*,6*H*-bis­([1,4]di­thio­cino)[6,7-*b*:6′,7′-*e*]pyrazine (**L**), with silver(I) nitrate leads to the formation of a two-dimensional coordination polymer, with the silver(I) atom coordinated by three S atoms of the ligand and an O atom of the nitrate anion (Assoumatine & Stoeckli-Evans, 2020 ▸). A series of pyrazine­thio­phanes, including ligand **L**, has been synthesized to study their coordination chemistry with transition metals (Assoumatine, 1999 ▸).

**Figure 1:**
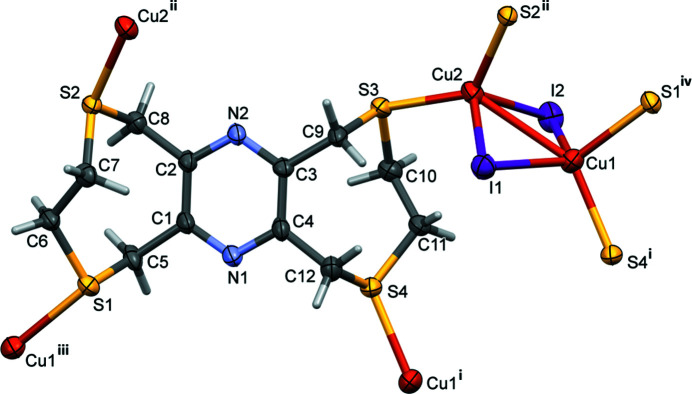
A view of the asymmetric unit of complex **I**, expanded to show the coordination environments of the two copper(I) atoms. Displacement ellipsoids are drawn at the 50% probability level. [Symmetry codes: (i) –*x*, –*y*, –*z*; (ii) –*x*, –*y* + 1, –*z*; (iii) *x* – 1, –*y* + 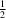 , *z* + 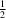 ; (iv) *x* + 1, –*y* + 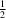 , *z* − 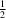 ].

The reaction of **L** with CuI leads to the formation of a two-dimensional coordination polymer, poly[[μ_4_-3,4,8,10,11,13-hexa­hydro-1*H*,6*H*-bis­([1,4]di­thio­cino)[6,7-*b*:6′,7′-*e*]pyrazine]di-μ-iodido-dicopper(I)], incorporating a [Cu_2_I_2_] motif (Fig. 1 ▸). The asymmetric unit is composed of a ligand mol­ecule, two copper(I) atoms and two I^−^ ions. The layers lie parallel to (102), and there are C—H⋯S and C—H⋯I intra­layer hydrogen bonds present (Table 1 ▸).

Selected bond lengths and bond angles involving the copper(I) atoms in **I** are given in Table 2 ▸. In **I**, both copper(I) atoms are considered to be fourfold S_2_I_2_ coordinate. The fourfold index parameter *τ*_4_ is 0.89 for atom Cu1 and 0.84 for atom Cu2 (*τ*_4_ = 1 for a perfect tetra­hedral environment, 0 for a perfect square-planar environment and 0.85 for a perfect trigonal–pyramidal environment; Yang *et al.*, 2007 ▸). Hence, both metal atoms have similar trigonal–pyramidal coordination environments. The distance Cu1⋯Cu2 is 2.7759 (11) Å. The Cu—S and Cu—I bond lengths involving atom Cu1 are noticeably different to those involving atom Cu2 (Table 2 ▸). Bond lengths Cu1—S1 and Cu1—S4 [2.3955 (16) and 2.3187 (16) Å, respectively] are longer than bond lengths Cu2—S2 and Cu2—S3 [2.3030 (16) and 2.3039 (16) Å, respectively]. In contrast, it can be seen that bond lengths Cu1—I1 and Cu1—I2 [2.6190 (10) and 2.5915 (10) Å, respectively] are shorter than bond lengths Cu2—I1 and Cu2—I2 [2.7117 (10) and 2.6460 (9) Å, respectively]. As in the silver nitrate complex of **L** mentioned above, the pyrazine N atoms are not involved in coordination to the copper(I) atoms.

In the complex, the ligand is step-shaped, as in the solid-state structure of the ligand itself (Assoumatine & Stoeckli-Evans, 2020 ▸). The conformation of the eight-membered rings fits best to the definition of a twist-boat-chair (Evans & Boeyens, 1988 ▸; Spek, 2020 ▸), with a *pseudo*-twofold rotation axis bis­ecting bonds C1—C2 and C6—C7 in one ring and bonds C3—C4 and C10—C11 in the second ring.

A search of the Cambridge Structural Database (CSD; Version 5.41, last update November 2019; Groom *et al.*, 2016 ▸) for the benzene analogue of **L**, or complexes of this analogue, gave no hits. A search for the S_2_CuI_2_CuS_2_ motif gave 34 hits for 33 structures (see file S1 in the supporting information). The Cu⋯Cu distances of the majority of these compounds vary from *ca* 2.580 to 3.087 Å (largest observed distance is 3.706 Å). For the majority of the compounds, the Cu—S bond lengths vary from 2.246 to 2.374 Å (largest observed distance is 2.531 Å), while the Cu—I bond lengths vary from 2.498 to 2.762 Å (largest observed bond length is 3.086 Å). It is evident from Table 2 ▸ that the bond lengths observed in complex **I** fall within these limits.

**Figure 2:**
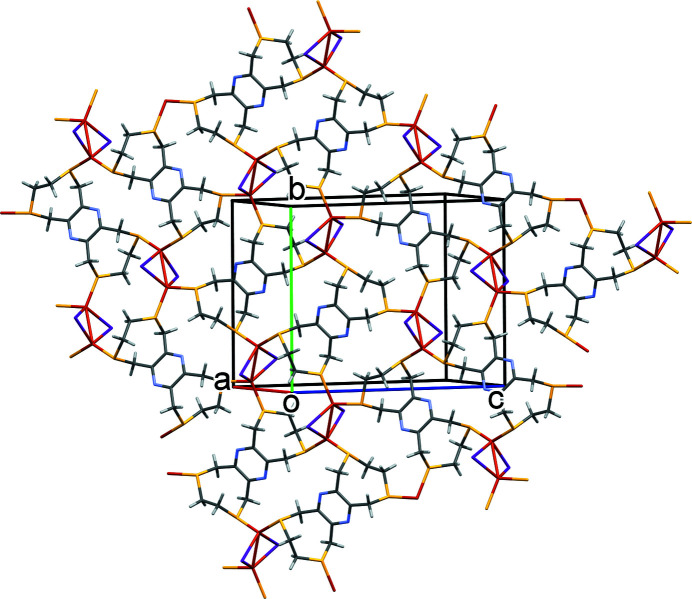
A view, almost normal to plane (102), of the crystal packing of complex **I**.

**Figure 3:**
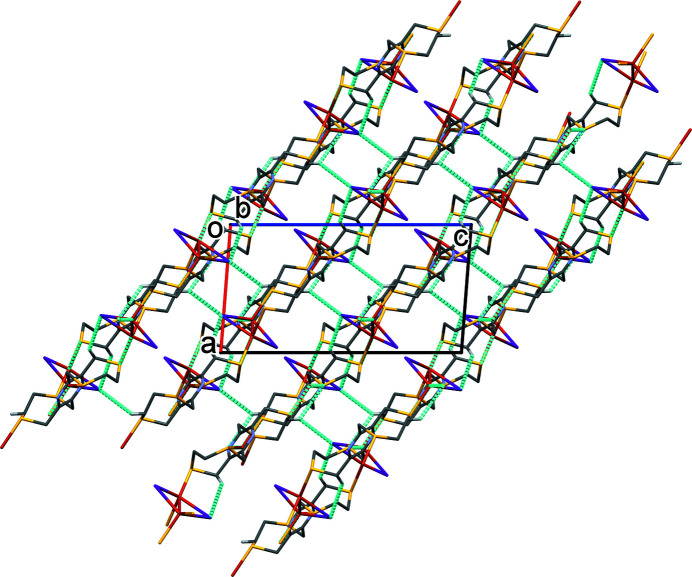
A view along the *b* axis of the crystal packing of complex **I**. Hydrogen bonds are shown as dashed lines (Table 1 ▸). For clarity, only the hydrogen atoms involved in these inter­actions have been included.

In the crystal of **I**, the layers lying parallel to plane (102) (Fig. 2 ▸) are linked by C—H⋯I hydrogen bonds forming a supra­molecular framework (Fig. 3 ▸ and Table 1 ▸). There are no other significant inter­molecular inter­actions present in the crystal.

## Synthesis and crystallization

The synthesis and crystal structure of the title ligand, 3,4,8,10,11,13-hexa­hydro-1*H*,6*H*-bis­([1,4]di­thio­cino)[6,7-*b*:6′,7′-*e*]pyrazine (**L**), have been reported (Assoumatine & Stoeckli-Evans, 2020 ▸).

**Synthesis of complex I:** A solution of **L** (20 mg, 0.06 mmol) in CHCl_3_ (10 ml) was introduced into a 16 mm diameter glass tube and layered with MeCN (2 ml) as a buffer zone. Then a solution of CuI (11 mg, 0.06 mmol) in MeCN (5 ml) was added very gently to avoid possible mixing. The glass tube was sealed under an atmosphere of nitro­gen and left in the dark at room temperature for at least 3 weeks, whereupon pale-yellow block-like crystals of complex **I** were isolated at the inter­face between the two solutions. Analysis for C_12_H_16_N_2_S_4_Cu_2_I_2_ (*M*_r_ = 697.46); calculated (%): C 20.66, H 2.32, N 4.02; found (%): C 20.90, H 2.31, N 3.93. The IR spectrum for **I** is shown in Fig. S1 of the supporting information.

## Refinement

Crystal data, data collection and structure refinement details are summarized in Table 3 ▸. The data were collected with a four-circle diffractometer at RT and only one equivalent of data were measured, hence *R*_int_ = 0.0. No suitable ψ scans could be found so the crystal was equated to a sphere and the *ABSSphere* absorption correction was applied (*PLATON*; Spek, 2020 ▸).

## Supplementary Material

Crystal structure: contains datablock(s) I, Global. DOI: 10.1107/S2414314620004678/wm4128sup1.cif

Structure factors: contains datablock(s) I. DOI: 10.1107/S2414314620004678/wm4128Isup2.hkl

Scheme for CSD search. DOI: 10.1107/S2414314620004678/wm4128sup3.tif

CSD Search. DOI: 10.1107/S2414314620004678/wm4128sup4.pdf

IR spectrum for complex I. DOI: 10.1107/S2414314620004678/wm4128sup5.tif

CCDC reference: 1994655

Additional supporting information:  crystallographic information; 3D view; checkCIF report

## Figures and Tables

**Table 1:** Hydrogen-bond geometry (Å, °)

*D*—H⋯*A*	*D*—H	H⋯*A*	*D*⋯*A*	*D*—H⋯*A*
C6—H6*A*⋯S4	0.97	2.80	3.409 (6)	122
C9—H9*A*⋯I1	0.97	2.99	3.771 (6)	138
C6—H6*A*⋯I1^i^	0.97	2.89	3.702 (6)	142

Symmetry code: (i) 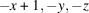 .

**Table 2:** Selected geometric parameters (Å, °)

Cu1—S1	2.3955 (16)	Cu2—S2	2.3030 (16)
Cu1—S4	2.3187 (16)	Cu2—S3	2.3039 (16)
Cu1—I1	2.6190 (9)	Cu2—I1	2.7117 (10)
Cu1—I2	2.5915 (10)	Cu2—I2	2.6460 (9)
Cu1—Cu2	2.7759 (11)		
			
S4—Cu1—S1	101.91 (6)	S2—Cu2—S3	129.92 (6)
S4—Cu1—I2	118.12 (5)	S2—Cu2—I2	108.76 (5)
S1—Cu1—I2	104.83 (5)	S3—Cu2—I2	106.63 (5)
S4—Cu1—I1	102.76 (5)	S2—Cu2—I1	90.49 (5)
S1—Cu1—I1	111.80 (5)	S3—Cu2—I1	107.95 (5)
I2—Cu1—I1	116.62 (3)	I2—Cu2—I1	111.68 (3)

**Table 3:** Experimental details

Crystal data	
Chemical formula	[Cu_2_I_2_(C_12_H_16_N_2_S_4_)]
*M*_r_	697.39
Crystal system, space group	Monoclinic, *P*2_1_/*c*
Temperature (K)	293
*a*, *b*, *c* (Å)	8.7612 (8), 13.1852 (13), 16.4458 (19)
β (°)	94.400 (9)
*V* (Å^3^)	1894.2 (3)
*Z*	4
Radiation type	Mo *K*α
μ (mm^−1^)	5.94
Crystal size (mm)	0.42 × 0.25 × 0.23

Data collection	
Diffractometer	STOE–Siemens AED2, 4-circle
Absorption correction	For a sphere (*ABSSphere*; Spek, 2020 ▸)
*T*_min_, *T*_max_	0.281, 0.295
No. of measured, independent and observed [*I* > 2σ(*I*)] reflections	3481, 3481, 3032
(sin θ/λ)_max_ (Å^−1^)	0.605

Refinement	
*R*[*F* ^2^ > 2σ(*F* ^2^)], *wR*(*F* ^2^), *S*	0.036, 0.084, 1.16
No. of reflections	3481
No. of parameters	199
H-atom treatment	H-atom parameters constrained
Δρ_max_, Δρ_min_ (e Å^−3^)	0.71, −0.69

Computer programs: *STADI4* and *X-RED* (Stoe & Cie, 1998 ▸), *SHELXS97* (Sheldrick, 2008 ▸), *SHELXL2016/6* (Sheldrick, 2015 ▸), *PLATON* (Spek, 2020 ▸), *Mercury* (Macrae *et al.*, 2020 ▸) and *publCIF* (Westrip, 2010 ▸).

## full crystallographic data

### Crystal data

**Table:**

[Cu_2_I_2_(C_12_H_16_N_2_S_4_)]	*F*(000) = 1320
*M**_r_* = 697.39	*D*_x_ = 2.445 Mg m^−^^3^
Monoclinic, *P*2_1_/*c*	Mo *K*α radiation, λ = 0.71073 Å
*a* = 8.7612 (8) Å	Cell parameters from 25 reflections
*b* = 13.1852 (13) Å	θ = 12.6–18.8°
*c* = 16.4458 (19) Å	µ = 5.94 mm^−^^1^
β = 94.400 (9)°	*T* = 293 K
*V* = 1894.2 (3) Å^3^	Block, yellow
*Z* = 4	0.42 × 0.25 × 0.23 mm

### Data collection

**Table:**

STOE–Siemens AED2, 4-circle diffractometer	3032 reflections with *I* > 2σ(*I*)
Radiation source: fine-focus sealed tube	*R*_int_ = 0.0000
Plane graphite monochromator	θ_max_ = 25.5°, θ_min_ = 2.5°
ω/\2q scans	*h* = −10→10
Absorption correction: for a sphere (*ABSSphere*; Spek, 2020)	*k* = 0→15
*T*_min_ = 0.281, *T*_max_ = 0.295	*l* = 0→19
3481 measured reflections	3 standard reflections every 60 min
3481 independent reflections	intensity decay: 2.5%

### Refinement

**Table:**

Refinement on *F*^2^	Primary atom site location: structure-invariant direct methods
Least-squares matrix: full	Secondary atom site location: difference Fourier map
*R*[*F*^2^ > 2σ(*F*^2^)] = 0.036	Hydrogen site location: inferred from neighbouring sites
*wR*(*F*^2^) = 0.084	H-atom parameters constrained
*S* = 1.16	*w* = 1/[σ^2^(*F*_o_^2^) + (0.0312*P*)^2^ + 6.7359*P*] where *P* = (*F*_o_^2^ + 2*F*_c_^2^)/3
3481 reflections	(Δ/σ)_max_ < 0.001
199 parameters	Δρ_max_ = 0.71 e Å^−^^3^
0 restraints	Δρ_min_ = −0.69 e Å^−^^3^

### Special details

**Table:**

Geometry. All esds (except the esd in the dihedral angle between two l.s. planes) are estimated using the full covariance matrix. The cell esds are taken into account individually in the estimation of esds in distances, angles and torsion angles; correlations between esds in cell parameters are only used when they are defined by crystal symmetry. An approximate (isotropic) treatment of cell esds is used for estimating esds involving l.s. planes.

### Fractional atomic coordinates and isotropic or equivalent isotropic displacement parameters (Å^2^)

**Table:**

	*x*	*y*	*z*	*U*_iso_*/*U*_eq_	
Cu1	0.25837 (9)	0.05716 (6)	−0.12025 (5)	0.03502 (19)	
Cu2	0.12846 (9)	0.24930 (6)	−0.12571 (5)	0.03483 (19)	
I1	0.29009 (5)	0.17469 (3)	0.00833 (2)	0.03617 (12)	
I2	0.04113 (5)	0.10364 (3)	−0.22976 (3)	0.03942 (13)	
S1	0.48581 (17)	0.05318 (11)	−0.19226 (9)	0.0303 (3)	
S2	0.34612 (17)	0.32435 (11)	−0.16802 (9)	0.0279 (3)	
S3	−0.09175 (18)	0.32212 (11)	−0.08436 (10)	0.0321 (3)	
S4	0.25769 (18)	−0.10386 (11)	−0.06361 (10)	0.0312 (3)	
N1	0.3515 (6)	−0.3124 (4)	−0.1619 (3)	0.0293 (11)	
N2	−0.2450 (5)	0.4626 (3)	0.0627 (3)	0.0269 (10)	
C1	0.5775 (7)	0.0972 (4)	−0.3460 (3)	0.0283 (13)	
C2	0.3672 (6)	0.5213 (4)	−0.1047 (3)	0.0250 (12)	
C3	−0.1754 (6)	0.3721 (4)	0.0703 (3)	0.0241 (12)	
C4	0.2279 (6)	−0.2971 (4)	−0.1210 (4)	0.0285 (13)	
C5	0.4370 (7)	0.0879 (5)	−0.2977 (4)	0.0312 (13)	
H5A	0.368915	0.036971	−0.322989	0.037*	
H5B	0.382704	0.152044	−0.299458	0.037*	
C6	0.5233 (7)	−0.0809 (4)	−0.2035 (4)	0.0304 (13)	
H6A	0.519170	−0.113186	−0.150759	0.036*	
H6B	0.442255	−0.109868	−0.239791	0.036*	
C7	0.3230 (7)	0.3938 (5)	−0.2633 (4)	0.0295 (13)	
H7A	0.262393	0.353567	−0.303239	0.035*	
H7B	0.267680	0.456170	−0.254845	0.035*	
C8	0.4350 (6)	0.4182 (4)	−0.0976 (4)	0.0276 (12)	
H8A	0.427790	0.394136	−0.042326	0.033*	
H8B	0.542830	0.422877	−0.106818	0.033*	
C9	−0.0402 (7)	0.3558 (4)	0.0216 (4)	0.0308 (13)	
H9A	0.022699	0.302154	0.046841	0.037*	
H9B	0.020761	0.417228	0.023067	0.037*	
C10	−0.2260 (7)	0.2176 (5)	−0.0786 (4)	0.0327 (13)	
H10A	−0.315614	0.243139	−0.054062	0.039*	
H10B	−0.258726	0.197287	−0.133864	0.039*	
C11	0.1717 (7)	−0.1229 (4)	0.0318 (4)	0.0290 (13)	
H11A	0.193515	−0.064180	0.066213	0.035*	
H11B	0.061486	−0.126864	0.020645	0.035*	
C12	0.1564 (7)	−0.1940 (4)	−0.1323 (4)	0.0325 (13)	
H12A	0.049539	−0.197057	−0.120952	0.039*	
H12B	0.161706	−0.172085	−0.188363	0.039*	

### Atomic displacement parameters (Å^2^)

**Table:**

	*U*^11^	*U*^22^	*U*^33^	*U*^12^	*U*^13^	*U*^23^
Cu1	0.0399 (4)	0.0265 (4)	0.0396 (4)	0.0031 (3)	0.0092 (3)	0.0012 (3)
Cu2	0.0321 (4)	0.0366 (4)	0.0377 (4)	−0.0002 (3)	0.0149 (3)	−0.0002 (3)
I1	0.0411 (2)	0.0373 (2)	0.0302 (2)	0.00574 (18)	0.00281 (17)	0.00025 (17)
I2	0.0331 (2)	0.0469 (3)	0.0380 (2)	0.00192 (19)	0.00058 (17)	−0.00704 (19)
S1	0.0302 (8)	0.0294 (8)	0.0332 (8)	0.0000 (6)	0.0142 (6)	−0.0026 (6)
S2	0.0321 (7)	0.0208 (7)	0.0325 (8)	−0.0023 (6)	0.0140 (6)	−0.0019 (6)
S3	0.0345 (8)	0.0267 (8)	0.0371 (8)	0.0040 (6)	0.0165 (6)	0.0062 (6)
S4	0.0356 (8)	0.0218 (7)	0.0382 (8)	−0.0004 (6)	0.0153 (7)	0.0020 (6)
N1	0.032 (3)	0.025 (3)	0.033 (3)	−0.001 (2)	0.014 (2)	−0.003 (2)
N2	0.031 (3)	0.022 (2)	0.030 (3)	−0.002 (2)	0.013 (2)	−0.006 (2)
C1	0.034 (3)	0.025 (3)	0.028 (3)	0.002 (3)	0.014 (2)	0.007 (2)
C2	0.026 (3)	0.022 (3)	0.028 (3)	−0.003 (2)	0.008 (2)	−0.006 (2)
C3	0.023 (3)	0.022 (3)	0.027 (3)	−0.005 (2)	0.006 (2)	−0.002 (2)
C4	0.027 (3)	0.026 (3)	0.033 (3)	0.000 (2)	0.011 (2)	−0.009 (2)
C5	0.024 (3)	0.035 (3)	0.037 (3)	0.005 (3)	0.015 (3)	0.010 (3)
C6	0.034 (3)	0.028 (3)	0.032 (3)	0.005 (3)	0.013 (3)	0.005 (3)
C7	0.030 (3)	0.031 (3)	0.028 (3)	−0.005 (3)	0.005 (2)	−0.005 (3)
C8	0.026 (3)	0.030 (3)	0.027 (3)	0.001 (2)	0.007 (2)	0.000 (2)
C9	0.031 (3)	0.022 (3)	0.041 (4)	−0.001 (2)	0.017 (3)	−0.003 (3)
C10	0.033 (3)	0.033 (3)	0.033 (3)	−0.001 (3)	0.009 (3)	0.003 (3)
C11	0.030 (3)	0.027 (3)	0.032 (3)	−0.002 (2)	0.011 (2)	0.000 (2)
C12	0.035 (3)	0.031 (3)	0.032 (3)	0.005 (3)	0.007 (3)	−0.003 (3)

### Geometric parameters (Å, º)

**Table:**

Cu1—S1	2.3955 (16)	C2—C8	1.485 (8)
Cu1—S4	2.3187 (16)	C3—C4^iii^	1.395 (8)
Cu1—I1	2.6190 (9)	C3—C9	1.495 (7)
Cu1—I2	2.5915 (10)	C4—C12	1.502 (8)
Cu1—Cu2	2.7759 (11)	C5—H5A	0.9700
Cu2—S2	2.3030 (16)	C5—H5B	0.9700
Cu2—S3	2.3039 (16)	C6—C7^i^	1.529 (8)
Cu2—I1	2.7117 (10)	C6—H6A	0.9700
Cu2—I2	2.6460 (9)	C6—H6B	0.9700
S1—C6	1.810 (6)	C7—H7A	0.9700
S1—C5	1.812 (6)	C7—H7B	0.9700
S2—C7	1.812 (6)	C8—H8A	0.9700
S2—C8	1.827 (6)	C8—H8B	0.9700
S3—C10	1.819 (6)	C9—H9A	0.9700
S3—C9	1.821 (6)	C9—H9B	0.9700
S4—C11	1.809 (6)	C10—C11^iii^	1.524 (8)
S4—C12	1.823 (6)	C10—H10A	0.9700
N1—C4	1.333 (7)	C10—H10B	0.9700
N1—C1^i^	1.346 (8)	C11—H11A	0.9700
N2—C2^ii^	1.334 (7)	C11—H11B	0.9700
N2—C3	1.341 (7)	C12—H12A	0.9700
C1—C2^i^	1.399 (8)	C12—H12B	0.9700
C1—C5	1.520 (7)		
			
S4—Cu1—S1	101.91 (6)	C1—C5—S1	112.3 (4)
S4—Cu1—I2	118.12 (5)	C1—C5—H5A	109.2
S1—Cu1—I2	104.83 (5)	S1—C5—H5A	109.2
S4—Cu1—I1	102.76 (5)	C1—C5—H5B	109.2
S1—Cu1—I1	111.80 (5)	S1—C5—H5B	109.1
I2—Cu1—I1	116.62 (3)	H5A—C5—H5B	107.9
S4—Cu1—Cu2	146.61 (5)	C7^i^—C6—S1	115.0 (4)
S1—Cu1—Cu2	111.01 (5)	C7^i^—C6—H6A	108.5
I2—Cu1—Cu2	58.95 (3)	S1—C6—H6A	108.5
I1—Cu1—Cu2	60.27 (3)	C7^i^—C6—H6B	108.5
S2—Cu2—S3	129.92 (6)	S1—C6—H6B	108.5
S2—Cu2—I2	108.76 (5)	H6A—C6—H6B	107.5
S3—Cu2—I2	106.63 (5)	C6^iv^—C7—S2	112.1 (4)
S2—Cu2—I1	90.49 (5)	C6^iv^—C7—H7A	109.2
S3—Cu2—I1	107.95 (5)	S2—C7—H7A	109.2
I2—Cu2—I1	111.68 (3)	C6^iv^—C7—H7B	109.2
S2—Cu2—Cu1	93.15 (5)	S2—C7—H7B	109.2
S3—Cu2—Cu1	136.16 (5)	H7A—C7—H7B	107.9
I2—Cu2—Cu1	57.04 (3)	C2—C8—S2	115.0 (4)
I1—Cu2—Cu1	57.00 (2)	C2—C8—H8A	108.5
Cu1—I1—Cu2	62.73 (3)	S2—C8—H8A	108.5
Cu1—I2—Cu2	64.00 (3)	C2—C8—H8B	108.5
C6—S1—C5	100.4 (3)	S2—C8—H8B	108.5
C6—S1—Cu1	103.67 (19)	H8A—C8—H8B	107.5
C5—S1—Cu1	108.86 (19)	C3—C9—S3	113.5 (4)
C7—S2—C8	102.7 (3)	C3—C9—H9A	108.9
C7—S2—Cu2	116.0 (2)	S3—C9—H9A	108.9
C8—S2—Cu2	114.97 (19)	C3—C9—H9B	108.9
C10—S3—C9	104.4 (3)	S3—C9—H9B	108.9
C10—S3—Cu2	105.0 (2)	H9A—C9—H9B	107.7
C9—S3—Cu2	103.4 (2)	C11^iii^—C10—S3	118.0 (4)
C11—S4—C12	103.4 (3)	C11^iii^—C10—H10A	107.8
C11—S4—Cu1	119.3 (2)	S3—C10—H10A	107.8
C12—S4—Cu1	111.3 (2)	C11^iii^—C10—H10B	107.8
C4—N1—C1^i^	118.2 (5)	S3—C10—H10B	107.8
C2^ii^—N2—C3	118.1 (5)	H10A—C10—H10B	107.2
N1^iv^—C1—C2^i^	120.8 (5)	C10^iii^—C11—S4	114.7 (4)
N1^iv^—C1—C5	114.1 (5)	C10^iii^—C11—H11A	108.6
C2^i^—C1—C5	125.1 (5)	S4—C11—H11A	108.6
N2^ii^—C2—C1^iv^	120.9 (5)	C10^iii^—C11—H11B	108.6
N2^ii^—C2—C8	116.0 (5)	S4—C11—H11B	108.6
C1^iv^—C2—C8	123.1 (5)	H11A—C11—H11B	107.6
N2—C3—C4^iii^	121.2 (5)	C4—C12—S4	109.5 (4)
N2—C3—C9	116.9 (5)	C4—C12—H12A	109.8
C4^iii^—C3—C9	121.9 (5)	S4—C12—H12A	109.8
N1—C4—C3^iii^	120.8 (5)	C4—C12—H12B	109.8
N1—C4—C12	114.9 (5)	S4—C12—H12B	109.8
C3^iii^—C4—C12	124.3 (5)	H12A—C12—H12B	108.2
			
C2^ii^—N2—C3—C4^iii^	−0.6 (8)	C7—S2—C8—C2	43.6 (5)
C2^ii^—N2—C3—C9	179.1 (5)	Cu2—S2—C8—C2	−83.2 (4)
C1^i^—N1—C4—C3^iii^	1.2 (9)	N2—C3—C9—S3	−80.9 (6)
C1^i^—N1—C4—C12	179.4 (5)	C4^iii^—C3—C9—S3	98.9 (6)
N1^iv^—C1—C5—S1	−83.4 (6)	C10—S3—C9—C3	−50.9 (5)
C2^i^—C1—C5—S1	96.8 (7)	Cu2—S3—C9—C3	−160.6 (4)
C6—S1—C5—C1	−77.1 (5)	C9—S3—C10—C11^iii^	−58.6 (5)
Cu1—S1—C5—C1	174.4 (4)	Cu2—S3—C10—C11^iii^	49.8 (5)
C5—S1—C6—C7^i^	76.2 (5)	C12—S4—C11—C10^iii^	77.0 (5)
Cu1—S1—C6—C7^i^	−171.3 (4)	Cu1—S4—C11—C10^iii^	−158.8 (4)
C8—S2—C7—C6^iv^	67.3 (5)	N1—C4—C12—S4	−83.0 (6)
Cu2—S2—C7—C6^iv^	−166.5 (3)	C3^iii^—C4—C12—S4	95.2 (6)
N2^ii^—C2—C8—S2	85.1 (6)	C11—S4—C12—C4	−82.9 (5)
C1^iv^—C2—C8—S2	−92.7 (6)	Cu1—S4—C12—C4	147.8 (4)

Symmetry codes: (i) −*x*+1, *y*−1/2, −*z*−1/2; (ii) −*x*, −*y*+1, −*z*; (iii) −*x*, −*y*, −*z*; (iv) −*x*+1, *y*+1/2, −*z*−1/2.

### Hydrogen-bond geometry (Å, º)

**Table:**

*D*—H···*A*	*D*—H	H···*A*	*D*···*A*	*D*—H···*A*
C6—H6*A*···S4	0.97	2.80	3.409 (6)	122
C9—H9*A*···I1	0.97	2.99	3.771 (6)	138
C6—H6*A*···I1^v^	0.97	2.89	3.702 (6)	142

Symmetry code: (v) −*x*+1, −*y*, −*z*.
