# Supplementary figures and images for: Poly[[μ4-3,4,8,10,11,13-hexa­hydro-1H,6H-bis­([1,4]di­thio­cino)[6,7-b:6′,7′-e]pyrazine]di-μ-iodido-dicopper(I)]: a two-dimensional copper(I) coordination polymer
Source: IUCrdata. 2020 Apr 7;5(Pt 4):x200467. doi: 10.1107/S2414314620004678 (PMC9462211; doi:10.1107/S2414314620004678)

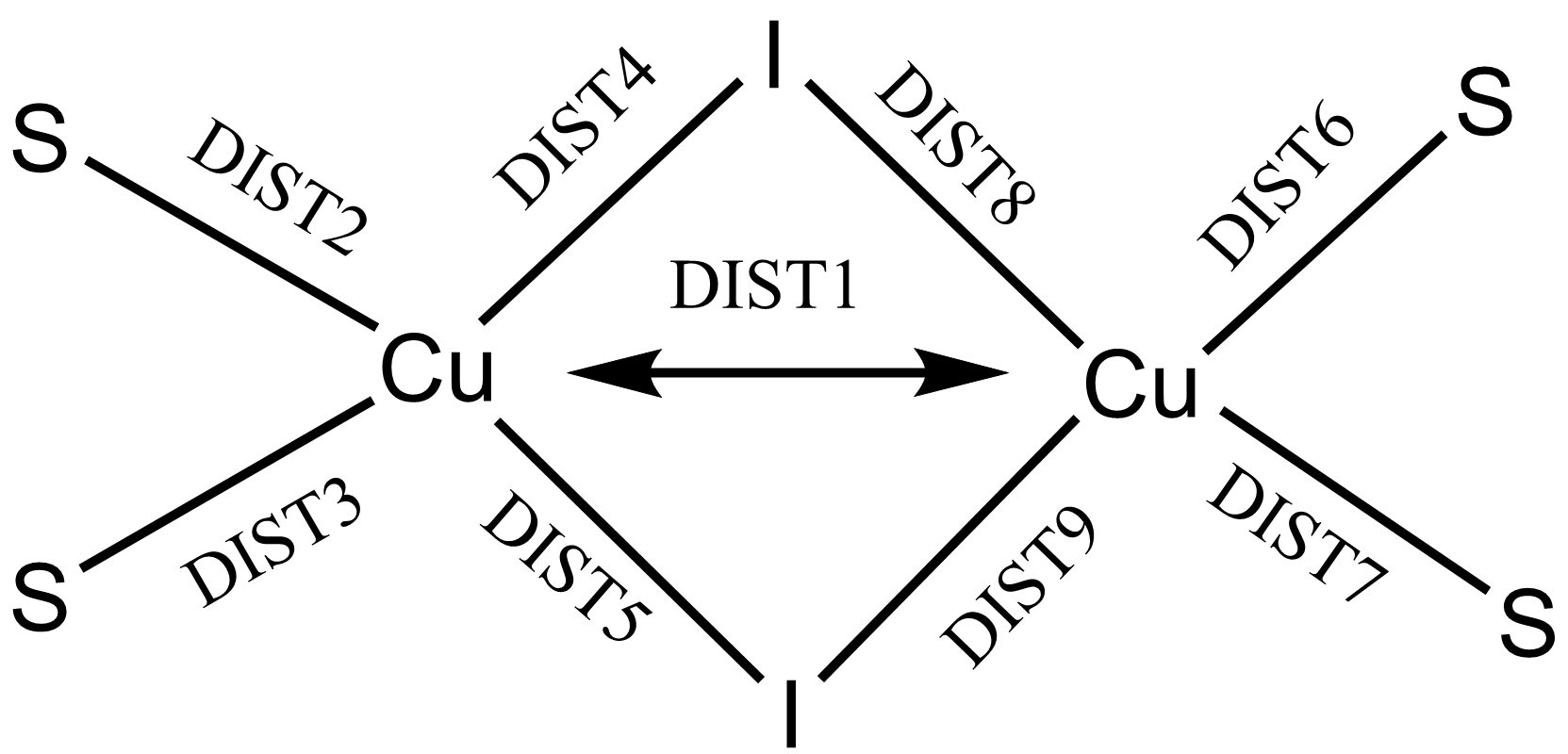

Supplement: Supplementary file 3 [file x-05-x200467-sup3.tif]

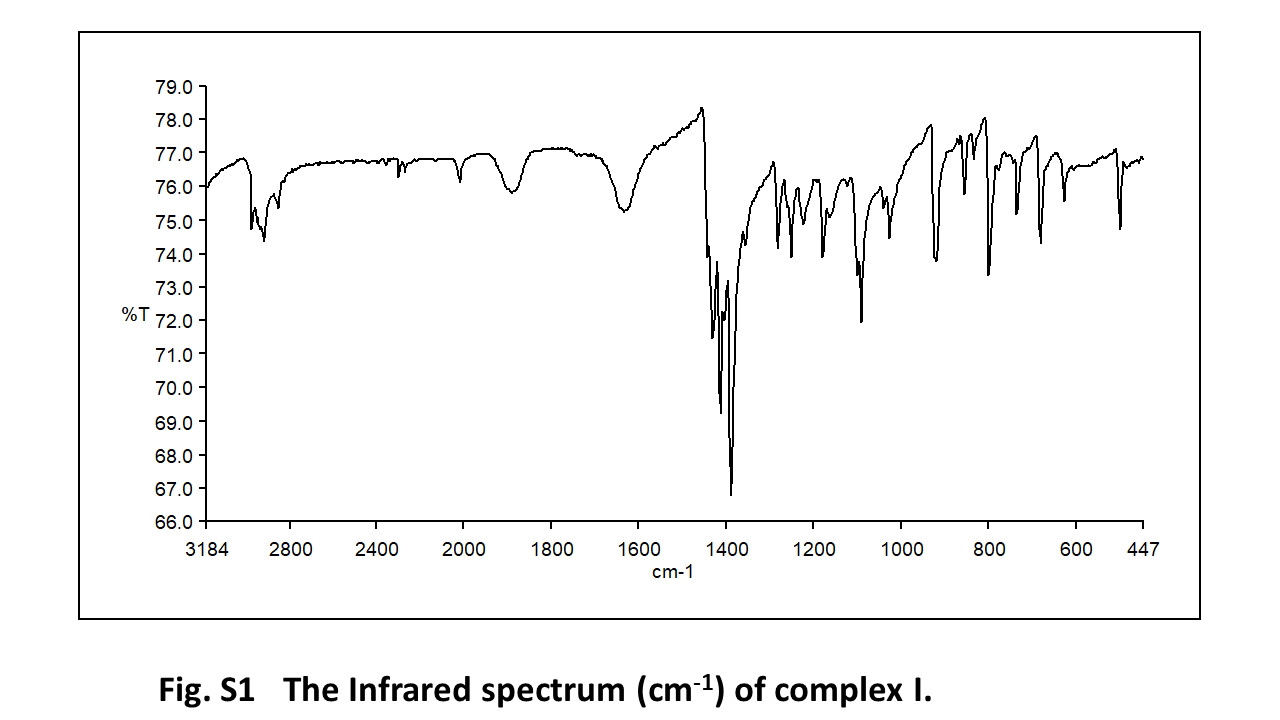

Supplement: Supplementary file 5 [file x-05-x200467-sup5.tif]
