# Supplementary material for: Poly[[μ4-3,4,8,10,11,13-hexa­hydro-1H,6H-bis­([1,4]di­thio­cino)[6,7-b:6′,7′-e]pyrazine]di-μ-iodido-dicopper(I)]: a two-dimensional copper(I) coordination polymer
Source: IUCrdata. 2020 Apr 7;5(Pt 4):x200467. doi: 10.1107/S2414314620004678 (PMC9462211; doi:10.1107/S2414314620004678)
Supplement: Supplementary file 4 [file x-05-x200467-sup4.pdf]

Search Overview

Search:	search2
Date/Time done:	Fri Mar 27 10:46:00 2020
Database(s):	CSD version 5.41 (November 2019)
Restriction Info:	No refcode restrictions applied
Filters:	None
Percentage Completed:	100%
Number of Hits:	34

Single query used. Search found structures that:

match

Query 1

Query 1

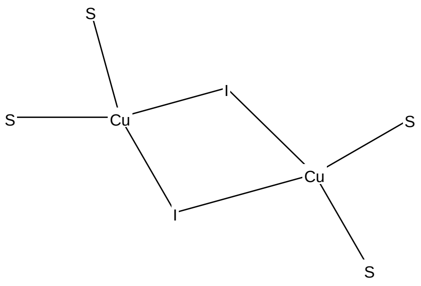

Search: search2 (Fri Mar 27 10:46:00 2020): Hits 1-4

BOBXUM

Reference: Tae Ho Kim, G.Park, Yong Woon Shin, K.-M.Park, Myong Yong Choi, Jineun Kim (2008) *Bull.Korean Chem.Soc.* ,**29,**499

Formula: $C_{40}\ H_{60}\ Cu_2\ I_2\ S_4$

Compound Name: bis((μ_2-Iodo)-(1,2-bis(cyclohexylthiomethyl)benzene))-di-copper(i)

Space Group:	P21/c	***Cell:***	***a*** 10.934(0)	***b*** 8.890(0)	***c*** 22.123(1)		
Space Group No.:	14	***(Å,°)***	α 90.00	β 97.30(0)	γ 90.00		
R-Factor (%):	2.39	***Temperature(K):***	173	***Density(g/cm³):***	1.635		

Parameters

Fragment 1

**DIST1 (D)**	2.956
**DIST2 (D)**	2.319
**DIST3 (D)**	2.320
**DIST4 (D)**	2.580
**DIST5 (D)**	2.728
**DIST6 (D)**	2.320
**DIST7 (D)**	2.319
**DIST8 (D)**	2.728
**DIST9 (D)**	2.580

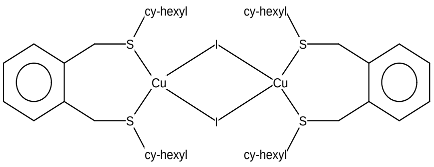

ENAWUM

Reference: P.R.Martinez-Alanis, V.M.Ugalde-Saldivar, I.Castillo (2011) *Eur.J.Inorg.Chem.* ,212

Formula: $C_{72}\ H_{102}\ Cu_2\ I_2\ N_2\ S_6$

Compound Name: bis(μ_2-Iodo)-bis(tris(((2-t-butyl-4-methylphenyl)thio)methyl)amine-S,S')-di-copper(i)

Space Group:	P21/c	***Cell:***	***a*** 16.630(2)	***b*** 14.794(2)	***c*** 15.185(2)		
Space Group No.:	14	***(Å,°)***	α 90.00	β 95.39(0)	γ 90.00		
R-Factor (%):	2.86	***Temperature(K):***	100	***Density(g/cm³):***	1.401		

Parameters

Fragment 1

**DIST1 (D)**	2.582
**DIST2 (D)**	2.338
**DIST3 (D)**	2.334
**DIST4 (D)**	2.658
**DIST5 (D)**	2.591
**DIST6 (D)**	2.334
**DIST7 (D)**	2.338
**DIST8 (D)**	2.591
**DIST9 (D)**	2.658

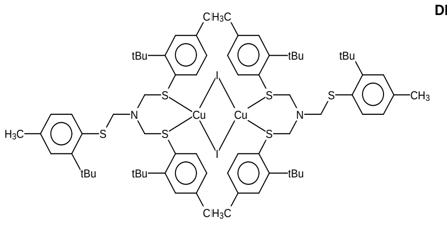

FUHCEQ

Reference: B.Noren, A.Oskarsson (1987) *Acta Chem.Scand.* ,**41,**12

Formula: $C_{16}\ H_{32}\ Cu_2\ I_2\ S_4$

Compound Name: bis(μ_2-Iodo)-bis(bis(tetrahydrothiophene)-copper(ii))

Space Group:	P21/n	***Cell:***	***a*** 10.629(3)	***b*** 10.577(2)	***c*** 11.291(2)		
Space Group No.:	14	***(Å,°)***	α 90.00	β 98.84(2)	γ 90.00		
R-Factor (%):	5.90	***Temperature(K):***	200	***Density(g/cm³):***	1.942		

Parameters

Fragment 1

**DIST1 (D)**	2.675
**DIST2 (D)**	2.318
**DIST3 (D)**	2.331
**DIST4 (D)**	2.637
**DIST5 (D)**	2.639
**DIST6 (D)**	2.331
**DIST7 (D)**	2.318
**DIST8 (D)**	2.639
**DIST9 (D)**	2.637

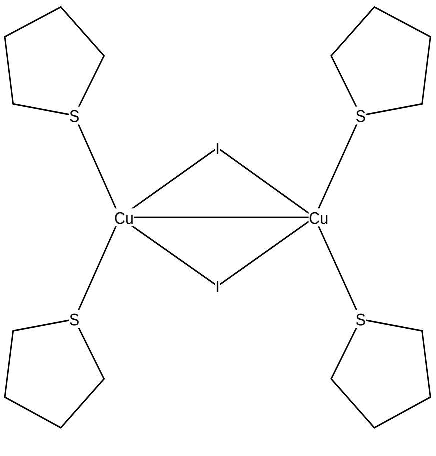

GIGCII

Reference: L.M.Engelhardt, P.C.Healy, B.W.Skelton, A.H.White (1988) *Aust.J.Chem.* ,**41,**839

Formula: $C_{42}\ H_{84}\ Co_2\ Cu_2\ I_2\ N_6\ S_{12}$

Compound Name: bis((μ_2-Iodo)-bis(μ_2-N,N-di-n-propyldithiocarbamato-S,S,S')-(N,N-di-n-propyldithiocarbamato-S,S')-cobalt(iii)-copper(i))

Space Group:	P21/c	***Cell:***	***a*** 13.722(7)	***b*** 16.854(7)	***c*** 16.787(8)		
Space Group No.:	14	***(Å,°)***	α 90.00	β 121.66(3)	γ 90.00		
R-Factor (%):	4.40	***Temperature(K):***	295	***Density(g/cm³):***	1.564		

Parameters

Fragment 1

**DIST1 (D)**	2.752
**DIST2 (D)**	2.417
**DIST3 (D)**	2.419
**DIST4 (D)**	2.575
**DIST5 (D)**	2.583
**DIST6 (D)**	2.419
**DIST7 (D)**	2.417
**DIST8 (D)**	2.583
**DIST9 (D)**	2.575

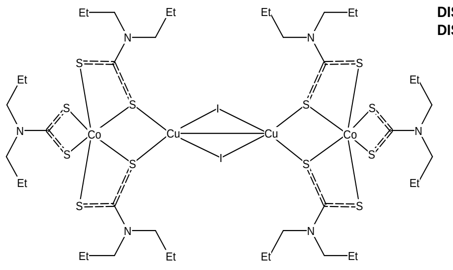

Search: search2 (Fri Mar 27 10:46:00 2020): Hits 5-8

HALZOK

Reference: M.Heller, W.S.Sheldrick (2004) *Z.Anorg.Allg.Chem.* ,**630,** 1869

Formula: $C_{12}\,H_{24}\,Cu_2\,I_2\,O_2\,S_4$

Compound Name: bis(μ_2-Iodo)-bis(1,4-dithia-7-oxacyclononane-1,4-diyl)-di-copper(i)

Space Group:	P21/n	***Cell:***	***a*** 7.535(1)	***b*** 17.897(4)	***c*** 8.044(4)		
Space Group No.:	14	***(Å,°)***	α 90.00	β 101.22(2)	γ 90.00		
R-Factor (%):	4.32	***Temperature(K):***	293	***Density(g/cm^3):***	2.215		

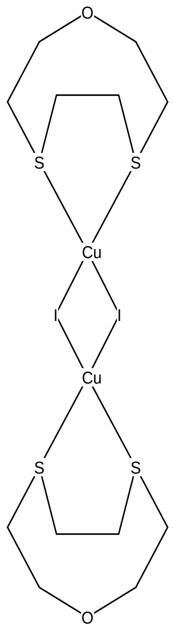

Parameters

Fragment 1

**DIST1 (D)**	2.860
**DIST2 (D)**	2.352
**DIST3 (D)**	2.372
**DIST4 (D)**	2.547
**DIST5 (D)**	2.710
**DIST6 (D)**	2.372
**DIST7 (D)**	2.352
**DIST8 (D)**	2.710
**DIST9 (D)**	2.547

HEZVAJ

Reference: Hong-wei Hou, Xin-quan Xin, Jie Liu, Ming-qin Chen, Shi Shu (1994) *J.Chem.Soc.,Dalton Trans.* ,3211

Formula: $4(C_8\,H_{20}\,N_1\,^{1+}),Br_2\,Cu_6\,I_4\,Mo_2\,O_2\,S_6\,^{4-}$

Compound Name: tetrakis(Tetraethylammonium) bis(μ_2-iodo)-bis(tris(μ_3-sulfido)-bromo-iodo-oxo-tri-copper-molybdenum)

Space Group:	C2/c	***Cell:***	***a*** 24.423(9)	***b*** 21.021(2)	***c*** 13.715(5)
Space Group No.:	15	***(Å,°)***	α 90.00	β 114.48(3)	γ 90.00
R-Factor (%):	6.70	***Temperature(K):***	295	***Density(g/cm^3):***	2.058

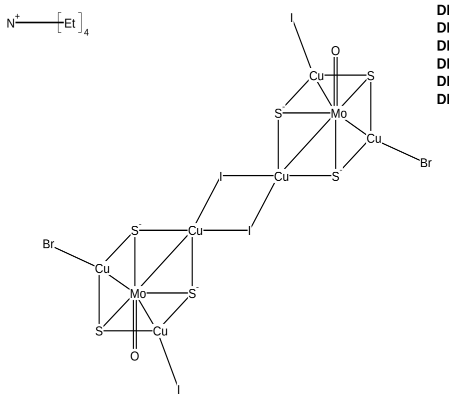

Parameters

Fragment 1

**DIST1 (D)**	3.356
**DIST2 (D)**	2.281
**DIST3 (D)**	2.278
**DIST4 (D)**	2.503
**DIST5 (D)**	2.972
**DIST6 (D)**	2.278
**DIST7 (D)**	2.281
**DIST8 (D)**	2.972
**DIST9 (D)**	2.503

HUWVOL

Reference: So Young Lee, Sunhong Park, Shim Sung Lee (2009) *Inorg.Chem.* ,**48,**11335

Formula: $C_{36}\,H_{40}\,Cu_2\,I_2\,O_4\,S_4$

Compound Name: bis(μ_2-Iodo)-bis(7,8,16,17-tetrahydro-5H,10H-dibenzo[e,m][1,4,8,11]dioxadithiacyclotetradecine-S,S')-di-copper(i)

Space Group:	P21/n	***Cell:***	***a*** 20.111(1)	***b*** 8.647(0)	***c*** 34.485(3)
Space Group No.:	14	***(Å,°)***	α 90.00	β 99.88(0)	γ 90.00
R-Factor (%):	5.08	***Temperature(K):***	173	***Density(g/cm^3):***	1.764

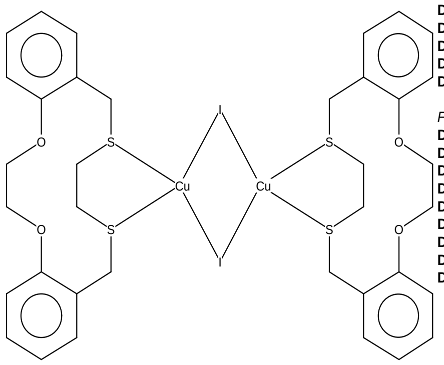

Parameters

Fragment 1

**DIST1 (D)**	2.688
**DIST2 (D)**	2.359
**DIST3 (D)**	2.335
**DIST4 (D)**	2.588
**DIST5 (D)**	2.627
**DIST6 (D)**	2.344
**DIST7 (D)**	2.347
**DIST8 (D)**	2.604
**DIST9 (D)**	2.594

Fragment 2

**DIST1 (D)**	2.762
**DIST2 (D)**	2.354
**DIST3 (D)**	2.330
**DIST4 (D)**	2.595
**DIST5 (D)**	2.603
**DIST6 (D)**	2.330
**DIST7 (D)**	2.354
**DIST8 (D)**	2.603
**DIST9 (D)**	2.595

IGOBIQ

Reference: Minhye Jo, J.Seo, L.F.Lindoy, Shim Sung Lee (2009) *Dalton Trans.* ,6096

Formula: $C_{40}\,H_{48}\,Cu_2\,I_2\,O_4\,S_6$

Compound Name: bis(μ_2-Iodo)-bis(2,5-dioxa-8,11,14-trithia-1,6(1,2)-dibenzenacyclopentadecaphane)-di-copper(i)

Space Group:	P-1	***Cell:***	***a*** 8.916(0)	***b*** 9.541(0)	***c*** 13.498(1)
Space Group No.:	2	***(Å,°)***	α 81.53(0)	β 80.15(0)	γ 77.86(0)
R-Factor (%):	3.51	***Temperature(K):***	173	***Density(g/cm^3):***	1.763

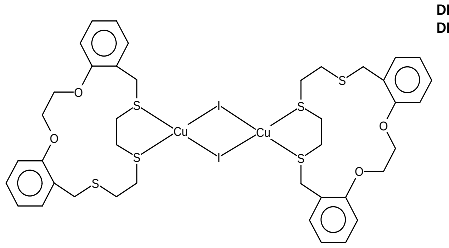

Parameters

Fragment 1

**DIST1 (D)**	2.683
**DIST2 (D)**	2.363
**DIST3 (D)**	2.330
**DIST4 (D)**	2.663
**DIST5 (D)**	2.589
**DIST6 (D)**	2.330
**DIST7 (D)**	2.363
**DIST8 (D)**	2.589
**DIST9 (D)**	2.663

Search: search2 (Fri Mar 27 10:46:00 2020): Hits 9-12

KAXHAT

Reference: B.Kure, S.Ogo, D.Inoki, H.Nakai, K.Isobe, S.Fukuzumi (2005) *J.Am.Chem.Soc.* ,**127,**14366

Formula: $C_{30}\,H_{54}\,Cu_4\,I_4\,Mo_2\,O_8\,P_2\,Ru_2\,S_6$

Compound Name: hexakis(μ_3-Sulfido)-bis(μ_2-iodo)-bis(iodo)-bis(η^6-hexamethylbenzene)-bis(trimethylphosphite)-tetra-copper-di-molybdenum-di-ruthenium

Space Group:	C2/c	***Cell:***	***a***	9.545(2)	***b***	18.288(3)	***c***	31.466(6)
Space Group No.:	15	***(Å,°)***	α	90.00	β	97.50(0)	γ	90.00

R-Factor (%): 4.16 ***Temperature(K):*** 173 ***Density(g/cm^3):*** 2.382

Parameters

Fragment 1

**DIST1 (D)**	3.317
**DIST2 (D)**	2.282
**DIST3 (D)**	2.297
**DIST4 (D)**	2.498
**DIST5 (D)**	3.046
**DIST6 (D)**	2.297
**DIST7 (D)**	2.282
**DIST8 (D)**	3.046
**DIST9 (D)**	2.498

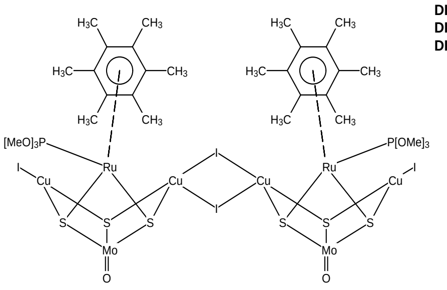

KAXJAV

Reference: B.Kure, S.Ogo, D.Inoki, H.Nakai, K.Isobe, S.Fukuzumi (2005) *J.Am.Chem.Soc.* ,**127,**14366

Formula: $C_{30}\,H_{54}\,Cu_4\,I_4\,Mo_2\,O_8\,P_2\,Ru_2\,S_6,2(C_3\,H_7\,N_1\,O_1)$

Compound Name: hexakis(μ_3-Sulfido)-bis(μ_2-iodo)-bis(iodo)-bis(η^6-hexamethylbenzene)-bis(trimethylphosphite)-tetra-copper-di-molybdenum-di-ruthenium dimethylformamide solvate

Space Group:	C2/c	***Cell:***	***a***	37.630(20)	***b***	9.293(4)	***c***	19.393(9)
Space Group No.:	15	***(Å,°)***	α	90.00	β	110.53(0)	γ	90.00

R-Factor (%): 5.34 ***Temperature(K):*** 173 ***Density(g/cm^3):*** 2.195

Parameters

Fragment 1

**DIST1 (D)**	3.530
**DIST2 (D)**	2.306
**DIST3 (D)**	2.290
**DIST4 (D)**	2.514
**DIST5 (D)**	2.972
**DIST6 (D)**	2.290
**DIST7 (D)**	2.306
**DIST8 (D)**	2.972
**DIST9 (D)**	2.514

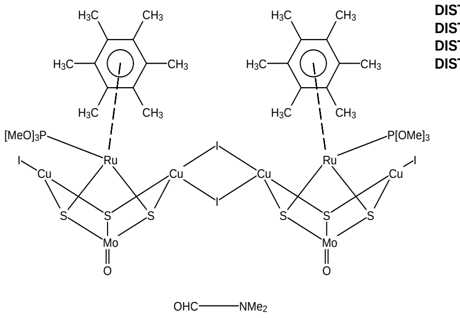

KEBREO

Reference: L.M.Engelhardt, P.C.Healy, B.W.Skelton, A.H.White (1989) *Aust.J.Chem.* ,**42,**885

Formula: $(C_{30}\,H_{60}\,Cr_2\,Cu_4\,I_4\,N_6\,S_{12})n,4n(C_2\,H_3\,N_1)$

Compound Name: catena-(bis(μ_2-Iodo)-tris(μ_3-N,N-diethyldithiocarbamato-S,S,S',S')-di-copper(i)-chromium(iii) acetonitrile solvate)

Space Group:	C2/c	***Cell:***	***a***	19.149(7)	***b***	14.814(4)	***c***	13.911(5)
Space Group No.:	15	***(Å,°)***	α	90.00	β	119.08(3)	γ	90.00

R-Factor (%): 3.70 ***Temperature(K):*** 295 ***Density(g/cm^3):*** 1.849

Parameters

Fragment 1

**DIST1 (D)**	2.931
**DIST2 (D)**	2.531
**DIST3 (D)**	2.375
**DIST4 (D)**	2.603
**DIST5 (D)**	2.573
**DIST6 (D)**	2.375
**DIST7 (D)**	2.531
**DIST8 (D)**	2.573
**DIST9 (D)**	2.603

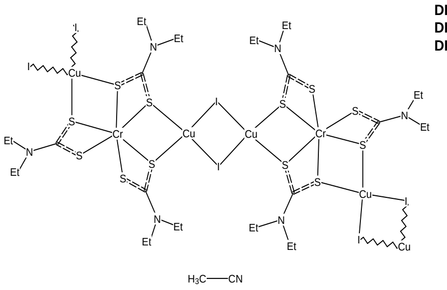

LAGSIY

Reference: J.Troyano, J.Perles, P.Amo-Ochoa, F.Zamora, S.Delgado (2016) *CrystEngComm* ,**18,**1809

Formula: $C_8\,H_{20}\,Cu_2\,I_2\,N_4\,S_4$

Compound Name: bis(μ_2-Iodo)-tetrakis(thioacetamide)-di-copper(i)

Space Group:	P-1	***Cell:***	***a***	7.429(0)	***b***	8.076(0)	***c***	9.614(0)
Space Group No.:	2	***(Å,°)***	α	107.06(0)	β	90.97(0)	γ	110.97(0)

R-Factor (%): 1.99 ***Temperature(K):*** 296 ***Density(g/cm^3):*** 2.218

Parameters

Fragment 1

**DIST1 (D)**	3.678
**DIST2 (D)**	2.246
**DIST3 (D)**	2.258
**DIST4 (D)**	2.637
**DIST5 (D)**	3.002
**DIST6 (D)**	2.258
**DIST7 (D)**	2.246
**DIST8 (D)**	3.002
**DIST9 (D)**	2.637

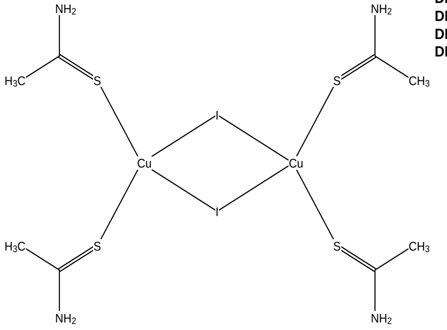

Search: search2 (Fri Mar 27 10:46:00 2020): Hits 13-16

MARFOB

Reference: Wen Lu, Zhe-Min Yan, Jie Dai, Yong Zhang, Qin-Yu Zhu, Ding-Xian Jia, Wen-Juan Guo (2005) *Eur.J.Inorg.Chem.* ,2339

Formula: $C_{24} H_{24} Cu_2 I_2 O_8 S_{12}$

Compound Name: bis((μ$_2$-Iodo)-(dimethyl 2-(4,5-bis(methylthio)-1,3-dithiol-2-ylidene)-1,3-dithiole-4,5-dicarboxylate))-di-copper(i)

Space Group:	P-1	***Cell:***	***a*** 7.981(3)	***b*** 8.394(4)	***c*** 15.107(6)
Space Group No.:	2	***(Å,°)***	α 86.77(1)	β 77.65(1)	γ 81.57(1)

R-Factor (%): 4.16 ***Temperature(K):*** 193 ***Density(g/cm³):*** 2.049

Parameters

Fragment 1

**DIST1 (D)**	2.647
**DIST2 (D)**	2.360
**DIST3 (D)**	2.350
**DIST4 (D)**	2.568
**DIST5 (D)**	2.603
**DIST6 (D)**	2.350
**DIST7 (D)**	2.360
**DIST8 (D)**	2.603
**DIST9 (D)**	2.568

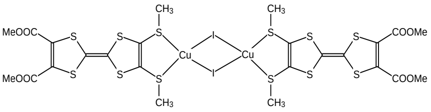

NEZBOJ

Reference: J.Ramos, V.M.Yartsev, S.Golhen, L.Ouahab, P.Delhaes (1997) *J.Mater.Chem.* ,**7,**1313

Formula: $C_{20} H_{20} Cu_2 I_2 S_{16}$

Compound Name: bis((μ$_2$-Iodo)-(ethylenedithio-bis(methylthio)tetrathiafulvalene)-copper)

Space Group:	C2	***Cell:***	***a*** 25.326(13)	***b*** 8.716(1)	***c*** 34.868(13)
Space Group No.:	5	***(Å,°)***	α 90.00	β 111.30(2)	γ 90.00

R-Factor (%): 5.75 ***Temperature(K):*** 295 ***Density(g/cm³):*** 2.138

Parameters

Fragment 1

**DIST1 (D)**	2.732
**DIST2 (D)**	2.399
**DIST3 (D)**	2.429
**DIST4 (D)**	2.625
**DIST5 (D)**	2.571
**DIST6 (D)**	2.351
**DIST7 (D)**	2.370
**DIST8 (D)**	2.586
**DIST9 (D)**	2.635

Fragment 2

**DIST1 (D)**	2.734
**DIST2 (D)**	2.392
**DIST3 (D)**	2.408
**DIST4 (D)**	2.626
**DIST5 (D)**	2.576
**DIST6 (D)**	2.361
**DIST7 (D)**	2.352
**DIST8 (D)**	2.591
**DIST9 (D)**	2.633

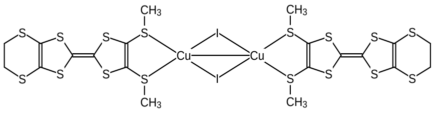

NEZBOJ01

Reference: R.E.Marsh, A.L.Spek (2001) *Acta Crystallogr.,Sect.B:Struct.Sci.* ,**57,**800

Formula: $C_{20} H_{20} Cu_2 I_2 S_{16}$

Compound Name: bis((μ$_2$-Iodo)-(ethylenedithio-bis(methylthio)tetrathiafulvalene)-copper)

Space Group:	Fdd2	***Cell:***	***a*** 64.972	***b*** 25.326	***c*** 8.716
Space Group No.:	43	***(Å,°)***	α 90.00	β 90.00	γ 90.00

R-Factor (%): 0.00 ***Temperature(K):*** 295 ***Density(g/cm³):*** 2.138

Parameters

Fragment 1

**DIST1 (D)**	2.733
**DIST2 (D)**	2.395
**DIST3 (D)**	2.419
**DIST4 (D)**	2.626
**DIST5 (D)**	2.573
**DIST6 (D)**	2.350
**DIST7 (D)**	2.368
**DIST8 (D)**	2.589
**DIST9 (D)**	2.633

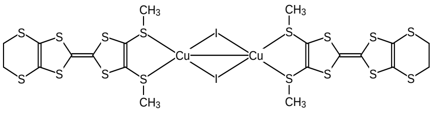

OCEHEM

Reference: J.K.Aulakh, T.S.Lobana, Henna Sood, D.S.Arora, I.Garcia-Santos, G.Hundal, M.Kaur, V.A.Smolenski, J.P.Jasinski (2017) *Dalton Trans.* ,**46,**1324

Formula: $C_{54} H_{60} Cu_4 I_4 N_{12} S_6$

Compound Name: bis(μ-iodo)-tetrakis(μ-1-phenylimidazolidine-2-thione)-diiodo-bis(1-phenylimidazolidine-2-thione)-tetra-copper(i)

Space Group:	P-1	***Cell:***	***a*** 9.573(4)	***b*** 10.198(5)	***c*** 16.821(8)
Space Group No.:	2	***(Å,°)***	α 100.18(2)	β 99.48(3)	γ 98.79(3)

R-Factor (%): 7.44 ***Temperature(K):*** 100 ***Density(g/cm³):*** 1.942

Parameters

Fragment 1

**DIST1 (D)**	2.765
**DIST2 (D)**	2.445
**DIST3 (D)**	2.326
**DIST4 (D)**	2.621
**DIST5 (D)**	2.690
**DIST6 (D)**	2.326
**DIST7 (D)**	2.445
**DIST8 (D)**	2.690
**DIST9 (D)**	2.621

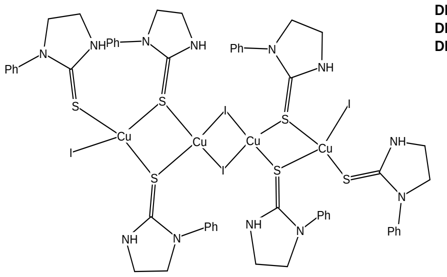

Search: search2 (Fri Mar 27 10:46:00 2020): Hits 17-20

PACHIN

Reference:	Yunji Kang, I.-H.Park, M.Ikeda, Y.Habata, Shim Sung Lee (2016) *Dalton Trans.* ,**45,**4528
Formula:	C_{116} H_{108} Cu_4 I_4 N_4 O_8 S_8,2(C_2 H_3 N_1),2(C_7 H_8)
Compound Name:	tetrakis(μ-iodo)-bis(μ-3,28,36,61-tetraoxa-11,20,44,53-tetrathia-67,68-diazanonacyclo[61.3.1.$1^{30,34}$.$0^{4,9}$.$0^{13,18}$.$0^{22,27}$.$0^{37,42}$.$0^{46,51}$.$0^{55,60}$]octahexaconta-1(67),4,6,8,13,15,17,22,24,26,30(68),31,33,37,39,41,46,48,50,55,57,59,63,65-tetracosaene)-tetra-copper acetonitrile toluene solvate

Space Group:	P-1	***Cell:***	***a*** 14.506(1)	***b*** 15.144(2)	***c*** 18.221(3)		
Space Group No.:	2	***(Å,°)***	α 98.41(1)	β 96.87(0)	γ 118.08(0)		
R-Factor (%):	14.37	***Temperature(K):***	173	***Density(g/cm³):***	1.446		

Parameters

Fragment 1

**DIST1 (D)**	2.965
**DIST2 (D)**	2.333
**DIST3 (D)**	2.345
**DIST4 (D)**	2.586
**DIST5 (D)**	2.698
**DIST6 (D)**	2.337
**DIST7 (D)**	2.311
**DIST8 (D)**	2.621
**DIST9 (D)**	2.685

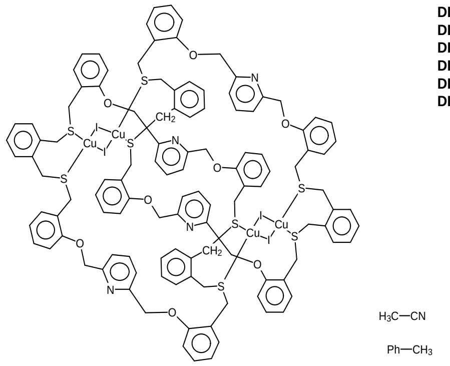

PACHOT

Reference:	Yunji Kang, I.-H.Park, M.Ikeda, Y.Habata, Shim Sung Lee (2016) *Dalton Trans.* ,**45,**4528
Formula:	C_{116} H_{108} Cu_4 I_4 N_4 O_8 S_8,2(C_7 H_8)
Compound Name:	tetrakis(μ-iodo)-bis(μ-2,28,36,61-tetraoxa-11,20,44,53-tetrathia-67,68-diazanonacyclo[61.3.1.$1^{30,34}$.$0^{4,9}$.$0^{13,18}$.$0^{22,27}$.$0^{37,42}$.$0^{46,51}$.$0^{55,60}$]octahexaconta-1(67),4,6,8,13,15,17,22,24,26,30(68),31,33,37,39,41,46,48,50,55,57,59,63,65-tetracosaene)-tetra-copper toluene solvate

Space Group:	P-1	***Cell:***	***a*** 14.275(3)	***b*** 14.778(3)	***c*** 18.504(3)		
Space Group No.:	2	***(Å,°)***	α 109.75(1)	β 93.68(1)	γ 116.30(1)		
R-Factor (%):	10.99	***Temperature(K):***	173	***Density(g/cm³):***	1.506		

Parameters

Fragment 1

**DIST1 (D)**	2.902
**DIST2 (D)**	2.317
**DIST3 (D)**	2.301
**DIST4 (D)**	2.573
**DIST5 (D)**	2.762
**DIST6 (D)**	2.336
**DIST7 (D)**	2.318
**DIST8 (D)**	2.598
**DIST9 (D)**	2.680

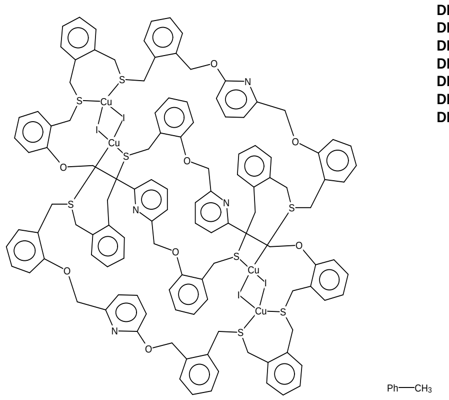

PIHPIH

Reference:	Hong Chen, Ting Cao, Yuqing Chen, Chunguang Chen, Zhongcheng Yue, Li Li, Yi Han, Yunyin Niu (2013) *Synth.React.Inorg.,Met.-Org.,Nano-Met.Chem.* ,**43,**1264
Formula:	2(C_{17} H_{24} N_2 $^{2+}$),Cu_6 I_6 Mo_2 O_2 S_6 $^{4-}$
Compound Name:	bis(1,1'-heptane-1,7-diyldipyridinium) hexakis(μ_3-sulfido)-bis(μ_2-iodo)-tetraiodo-dioxo-di-molybdenum-hexa-copper

Space Group:	P-1	***Cell:***	***a*** 11.098(1)	***b*** 11.849(1)	***c*** 12.234(1)		
Space Group No.:	2	***(Å,°)***	α 99.41(0)	β 107.12(0)	γ 109.21(0)		
R-Factor (%):	5.28	***Temperature(K):***	291	***Density(g/cm³):***	2.475		

Parameters

Fragment 1

**DIST1 (D)**	3.640
**DIST2 (D)**	2.263
**DIST3 (D)**	2.283
**DIST4 (D)**	2.500
**DIST5 (D)**	3.001
**DIST6 (D)**	2.283
**DIST7 (D)**	2.263
**DIST8 (D)**	3.001
**DIST9 (D)**	2.500

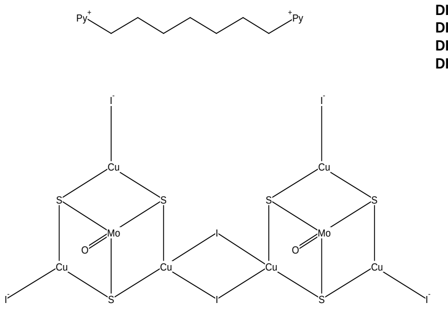

POFZER

Reference:	Hyunsoo Ryu, K.-M.Park, M.Ikeda, Y.Habata, Shim Sung Lee (2014) *Inorg.Chem.* ,**53,**4029
Formula:	C_{44} H_{56} Cu_2 I_2 O_8 S_4
Compound Name:	bis(μ_2-iodo)-bis(7,8,10,11,19,20,22,23-octahydro-5H,13H-dibenzo[h,s][1,4,7,14,11,17]tetraoxadithiacycloicosine)-di-copper(i)

Space Group:	P21/c	***Cell:***	***a*** 9.190(0)	***b*** 20.037(1)	***c*** 15.120(0)		
Space Group No.:	14	***(Å,°)***	α 90.00	β 120.88(0)	γ 90.00		
R-Factor (%):	2.03	***Temperature(K):***	173	***Density(g/cm³):***	1.698		

Parameters

Fragment 1

**DIST1 (D)**	2.846
**DIST2 (D)**	2.323
**DIST3 (D)**	2.325
**DIST4 (D)**	2.738
**DIST5 (D)**	2.587
**DIST6 (D)**	2.325
**DIST7 (D)**	2.323
**DIST8 (D)**	2.587
**DIST9 (D)**	2.738

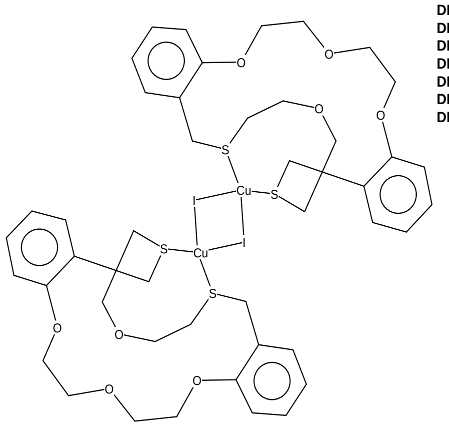

Search: search2 (Fri Mar 27 10:46:00 2020): Hits 21-24

PUGQIR

Reference:	Y.Suenaga, M.Maekawa, T.Kuroda-Sowa, M.Munakata, H.Morimoto, N.Hiyama, S.Kitagawa (1997) *Anal.Sci.* ,**13**,651
Formula:	C_{20} H_{28} Cu_2 I_2 S_8
Compound Name:	bis((μ_2-Iodo)-(1,2,4,5-tetramethylmercaptobenzene)-copper(i))

Space Group:	P-1	***Cell:***	***a*** 9.131(3)	***b*** 10.235(3)	***c*** 8.130(3)
Space Group No.:	2	***(Å,°)***	α 92.25	β 102.20	γ 91.10
R-Factor (%):	3.10	***Temperature(K):*** 295	***Density(g/cm³):*** 2.028		

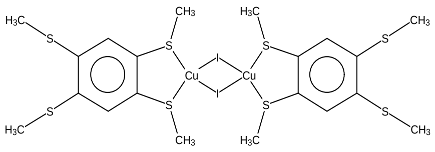

Parameters

Fragment 1

**DIST1 (D)**	2.616
**DIST2 (D)**	2.345
**DIST3 (D)**	2.342
**DIST4 (D)**	2.618
**DIST5 (D)**	2.602
**DIST6 (D)**	2.342
**DIST7 (D)**	2.345
**DIST8 (D)**	2.602
**DIST9 (D)**	2.618

QAVKOQ

Reference:	Dan Wang, Su-Yun Wu, Hai-Pu Li, Ying Yang, H.W.Roesky (2017) *Eur.J.Inorg.Chem.* ,1406
Formula:	C_{96} H_{128} Cu_2 I_2 N_8 O_4 S_4
Compound Name:	tetrakis(4-t-butyl-N-((2,6-diisopropylphenyl)carbamothioyl)benzamide)-bis(μ-iodo)-di-copper(i)

Space Group:	P-1	***Cell:***	***a*** 12.684(0)	***b*** 14.494(0)	***c*** 14.647(0)
Space Group No.:	2	***(Å,°)***	α 84.36(0)	β 71.84(0)	γ 86.23(0)
R-Factor (%):	4.08	***Temperature(K):*** 296	***Density(g/cm³):*** 1.284		

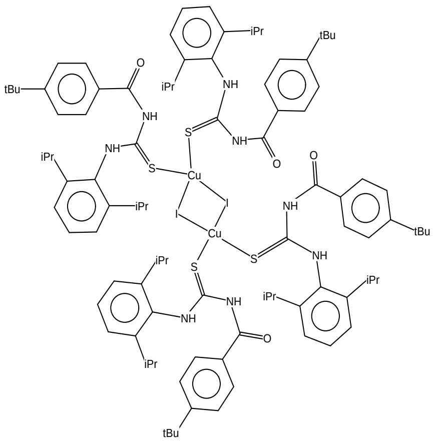

Parameters

Fragment 1

**DIST1 (D)**	2.878
**DIST2 (D)**	2.345
**DIST3 (D)**	2.299
**DIST4 (D)**	2.594
**DIST5 (D)**	2.685
**DIST6 (D)**	2.299
**DIST7 (D)**	2.345
**DIST8 (D)**	2.685
**DIST9 (D)**	2.594

QAVLOR

Reference:	Dan Wang, Su-Yun Wu, Hai-Pu Li, Ying Yang, H.W.Roesky (2017) *Eur.J.Inorg.Chem.* ,1406
Formula:	C_{96} H_{104} Cu_2 I_2 N_8 O_4 S_4
Compound Name:	tetrakis(N-((2,6-diisopropylphenyl)carbamothioyl)-1-naphthamide)-bis(μ-iodo)-di-copper(i)

Space Group:	P-1	***Cell:***	***a*** 12.631(0)	***b*** 13.706(0)	***c*** 14.831(0)
Space Group No.:	2	***(Å,°)***	α 80.16(0)	β 76.12(0)	γ 69.54(0)
R-Factor (%):	2.60	***Temperature(K):*** 293	***Density(g/cm³):*** 1.388		

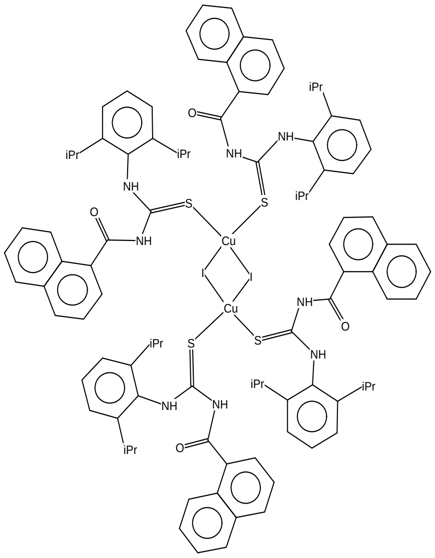

Parameters

Fragment 1

**DIST1 (D)**	3.076
**DIST2 (D)**	2.295
**DIST3 (D)**	2.343
**DIST4 (D)**	2.607
**DIST5 (D)**	2.672
**DIST6 (D)**	2.343
**DIST7 (D)**	2.295
**DIST8 (D)**	2.672
**DIST9 (D)**	2.607

QUPXAC

Reference:	A.Bonnot, M.Knorr, C.Strohmann, C.Golz, D.Fortin, P.D.Harvey (2015) *J.Inorg.Organomet.Polym.Mater.* ,**25**,480
Formula:	C_{36} H_{36} Cu_2 I_2 S_4
Compound Name:	bis(μ_2-(1,1'-(But-2-yne-1,4-diylbis((sulfanediyl)methylene))dibenzene))-bis(μ_2-iodo)-di-copper(i)

Space Group:	P21/c	***Cell:***	***a*** 13.135(0)	***b*** 9.289(0)	***c*** 15.395(0)
Space Group No.:	14	***(Å,°)***	α 90.00	β 106.65(0)	γ 90.00
R-Factor (%):	1.68	***Temperature(K):*** 173	***Density(g/cm³):*** 1.805		

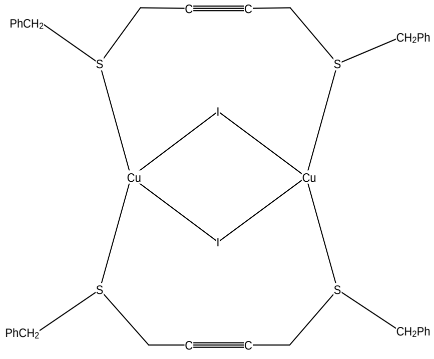

Parameters

Fragment 1

**DIST1 (D)**	2.928
**DIST2 (D)**	2.337
**DIST3 (D)**	2.316
**DIST4 (D)**	2.604
**DIST5 (D)**	2.648
**DIST6 (D)**	2.316
**DIST7 (D)**	2.337
**DIST8 (D)**	2.648
**DIST9 (D)**	2.604

Search: search2 (Fri Mar 27 10:46:00 2020): Hits 25-28

RIGZOW

Reference: L.I.Victoriano, M.T.Garland, A.Vega (1997) *Inorg.Chem.* , **36,**688

Formula: $C_{12}H_{24}Cu_2I_2N_4S_6$

Compound Name: bis((μ_2-Iodo)-(bis(N,N-dimethylthiocarbamoyl)sulfido)-copper(i))

Space Group:	P21/n	***Cell:***	***a*** 9.570(1)	***b*** 10.578(1)	***c*** 12.422(1)		
Space Group No.:	14	***(Å,°)***	α 90.00	β 104.42(1)	γ 90.00		
R-Factor (%):	3.70	***Temperature(K):***	295	***Density(g/cm3):***	2.175		

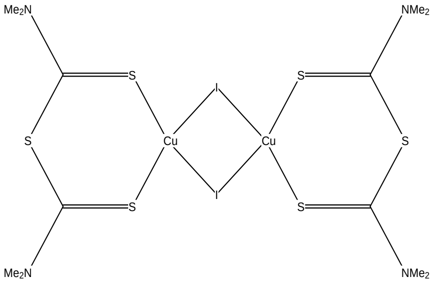

Parameters

Fragment 1

**DIST1 (D)**	2.985
**DIST2 (D)**	2.417
**DIST3 (D)**	2.332
**DIST4 (D)**	2.641
**DIST5 (D)**	2.646
**DIST6 (D)**	2.332
**DIST7 (D)**	2.417
**DIST8 (D)**	2.646
**DIST9 (D)**	2.641

SIRYOI

Reference: E.W.Ainscough, A.M.Brodie, A.Derwahl, G.H.Freeman, C.A.Otter (2007) *Polyhedron* ,**26,**5398

Formula: $C_{48}H_{44}Cu_2I_2N_4P_4S_4$,2($C_2H_3N_1$)

Compound Name: bis(μ_2-Iodo)-bis(μ_2-1,2-bis(diphenylthioylphosphino)hydrazine)-di-copper(i) acetonitrile solvate

Space Group:	P21/n	***Cell:***	***a*** 12.093(0)	***b*** 15.496(0)	***c*** 15.072(0)		
Space Group No.:	14	***(Å,°)***	α 90.00	β 94.65(0)	γ 90.00		
R-Factor (%):	1.98	***Temperature(K):***	200	***Density(g/cm3):***	1.642		

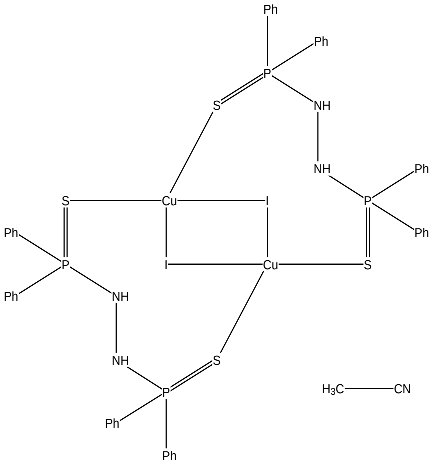

Parameters

Fragment 1

**DIST1 (D)**	3.706
**DIST2 (D)**	2.295
**DIST3 (D)**	2.304
**DIST4 (D)**	2.599
**DIST5 (D)**	3.086
**DIST6 (D)**	2.304
**DIST7 (D)**	2.295
**DIST8 (D)**	3.086
**DIST9 (D)**	2.599

TAKWIM

Reference: H.W.Yim, D.Rabinovich, K.-C.Lam, J.A.Golen, A.L.Rheingold (2003) *Acta Crystallogr.,Sect.E:Struct.Rep.Online* ,**59,** m556

Formula: $C_{14}H_{36}Cu_2I_2S_6Si_2$

Compound Name: bis(μ_2-Iodo)-bis(methyltris(methylthiomethyl)silane-κ^2S,S')-di-copper(i)

Space Group:	P21/c	***Cell:***	***a*** 9.337(0)	***b*** 12.835(0)	***c*** 13.099(0)		
Space Group No.:	14	***(Å,°)***	α 90.00	β 103.32(0)	γ 90.00		
R-Factor (%):	4.20	***Temperature(K):***	173	***Density(g/cm3):***	1.813		

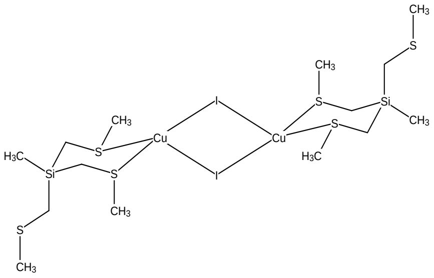

Parameters

Fragment 1

**DIST1 (D)**	2.863
**DIST2 (D)**	2.305
**DIST3 (D)**	2.322
**DIST4 (D)**	2.580
**DIST5 (D)**	2.692
**DIST6 (D)**	2.322
**DIST7 (D)**	2.305
**DIST8 (D)**	2.692
**DIST9 (D)**	2.580

TIYZEJ

Reference: J.Troyano, E.Zapata, J.Perles, P.Amo-Ochoa, V.Fernandez-Moreira, J.I.Martinez, F.Zamora, S.Delgado (2019) *Inorg.Chem.* ,**58,**3290

Formula: $C_{28}H_{28}Cu_2I_2N_4S_4$,2($C_2H_3N_1$)

Compound Name: tetrakis(benzenecarbothioamide)-bis(μ-iodo)-di-copper(i) acetonitrile solvate

Space Group:	P21/c	***Cell:***	***a*** 12.245(0)	***b*** 7.387(0)	***c*** 23.055(0)		
Space Group No.:	14	***(Å,°)***	α 90.00	β 104.40(0)	γ 90.00		
R-Factor (%):	2.62	***Temperature(K):***	296	***Density(g/cm3):***	1.664		

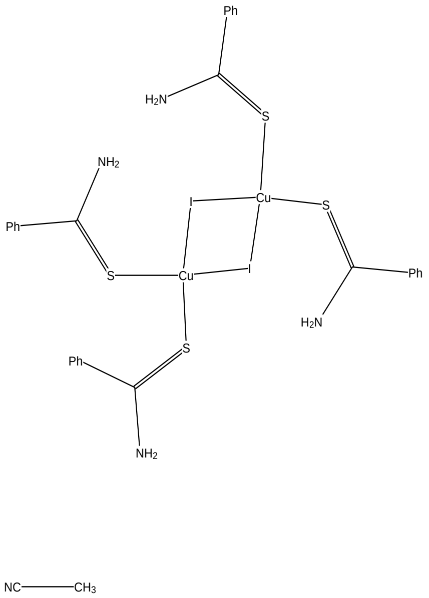

Parameters

Fragment 1

**DIST1 (D)**	3.379
**DIST2 (D)**	2.276
**DIST3 (D)**	2.276
**DIST4 (D)**	2.672
**DIST5 (D)**	2.724
**DIST6 (D)**	2.276
**DIST7 (D)**	2.276
**DIST8 (D)**	2.724
**DIST9 (D)**	2.672

Search: search2 (Fri Mar 27 10:46:00 2020): Hits 29-32

TIYZEJ01

Reference:	J.Troyano, E.Zapata, J.Perles, P.Amo-Ochoa, V.Fernandez-Moreira, J.I.Martinez, F.Zamora, S.Delgado (2019) *Inorg.Chem.* ,**58,**3290
Formula:	$C_{28} H_{28} Cu_2 I_2 N_4 S_4,2(C_2 H_3 N_1)$
Compound Name:	tetrakis(benzenecarbothioamide)-bis(μ-iodo)-di-copper acetonitrile solvate

Space Group:	P21/c	***Cell:***	***a*** 12.192(0)	***b*** 7.251(0)	***c*** 22.675(0)		
Space Group No.:	14	***(Å,°)***	α 90.00	β 103.15(0)	γ 90.00		
R-Factor (%):	1.69	***Temperature(K):***	110	***Density(g/cm³):***	1.721		

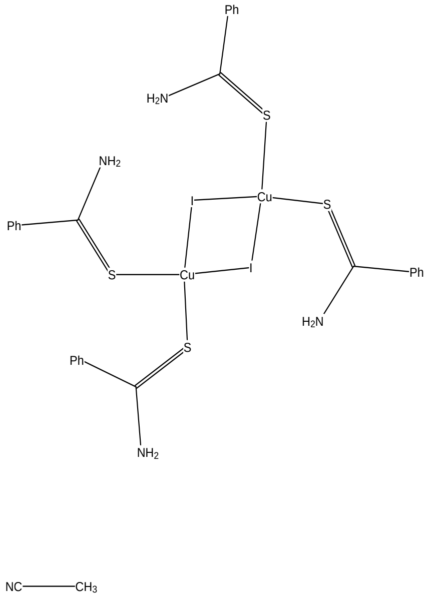

Parameters

Fragment 1

**DIST1 (D)**	3.320
**DIST2 (D)**	2.277
**DIST3 (D)**	2.276
**DIST4 (D)**	2.663
**DIST5 (D)**	2.699
**DIST6 (D)**	2.276
**DIST7 (D)**	2.277
**DIST8 (D)**	2.699
**DIST9 (D)**	2.663

TOJYOG

Reference:	Hongwei Hou, Deliang Long, Xinquan Xin, Xiaoxing Huang, Beisheng Kang, Ping Ge, Wei Ji, Shu Shi (1996) *Inorg.Chem.* ,**35,**5363
Formula:	$4(C_8 H_{20} N_1\ ^{1+}),Cu_6 I_6 Mo_2 O_2 S_6\ ^{4-}$
Compound Name:	tetrakis(Tetraethylammonium) hexaiodo-dioxo-hexasulfido-hexa-copper-di-molybdenum

Space Group:	C2/c	***Cell:***	***a*** 24.680(1)	***b*** 21.328(5)	***c*** 13.638(4)		
Space Group No.:	15	***(Å,°)***	α 90.00	β 114.51(3)	γ 90.00		
R-Factor (%):	4.30	***Temperature(K):***	295	***Density(g/cm³):***	2.115		

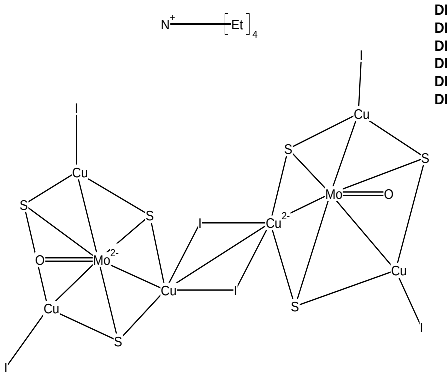

Parameters

Fragment 1

**DIST1 (D)**	3.386
**DIST2 (D)**	2.289
**DIST3 (D)**	2.286
**DIST4 (D)**	2.517
**DIST5 (D)**	2.984
**DIST6 (D)**	2.286
**DIST7 (D)**	2.289
**DIST8 (D)**	2.984
**DIST9 (D)**	2.517

UJUWIG

Reference:	Eun-Ju Kang, So Young Lee, Hayan Lee, Shim Sung Lee (2010) *Inorg.Chem.* ,**49,**7510
Formula:	$C_{52} H_{56} Cu_2 I_2 O_6 S_4,C_1 H_2 Cl_2$
Compound Name:	bis(μ2-Iodo)-bis(3,14,25-trioxa-11,17-dithiatetracyclo[25.3.1.0^4,9^.0^19,24^]hentriaconta-1(31),4,6,8,19,21,23,27,29-nonaene)-di-copper(i) dichloromethane solvate

Space Group:	P-1	***Cell:***	***a*** 10.659(0)	***b*** 13.294(0)	***c*** 20.890(1)		
Space Group No.:	2	***(Å,°)***	α 73.69(0)	β 76.23(0)	γ 72.91(0)		
R-Factor (%):	3.47	***Temperature(K):***	164	***Density(g/cm³):***	1.701		

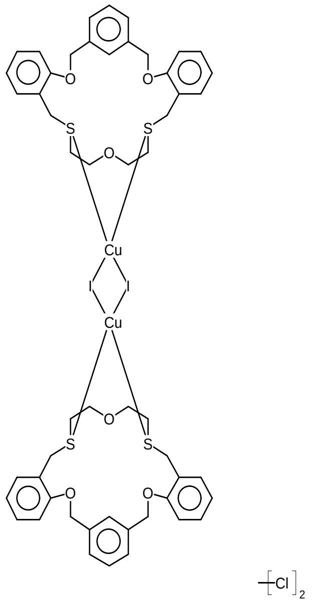

Parameters

Fragment 1

**DIST1 (D)**	2.701
**DIST2 (D)**	2.303
**DIST3 (D)**	2.291
**DIST4 (D)**	2.706
**DIST5 (D)**	2.638
**DIST6 (D)**	2.291
**DIST7 (D)**	2.303
**DIST8 (D)**	2.638
**DIST9 (D)**	2.706

Fragment 2

**DIST1 (D)**	2.802
**DIST2 (D)**	2.322
**DIST3 (D)**	2.316
**DIST4 (D)**	2.660
**DIST5 (D)**	2.626
**DIST6 (D)**	2.316
**DIST7 (D)**	2.322
**DIST8 (D)**	2.626
**DIST9 (D)**	2.660

WINLUC

Reference:	Li-Xia Shen, Xing-Hui Li, Huan-Huan Wu, Zhong-Cheng Yue, Li Li, Zhen-Hua Zhang, Yun-Yin Niu (2013) *Synth.React.Inorg.,Met.-Org.,Nano-Met.Chem.* ,**43,**1372
Formula:	$4(C_{10} H_{24} N_1\ ^{1+}),Cu_6 I_6 Mo_2 O_2 S_6\ ^{4-}$
Compound Name:	tetrakis(Triethyl(n-butyl)ammonium) hexakis(μ3-sulfido)-bis(μ2-iodo)-tetrakis(iodo)-dioxo-hexa-copper-di-molybdenum

Space Group:	P-1	***Cell:***	***a*** 11.911(1)	***b*** 12.812(1)	***c*** 13.019(1)		
Space Group No.:	2	***(Å,°)***	α 66.97(0)	β 77.74(0)	γ 88.47(0)		
R-Factor (%):	3.25	***Temperature(K):***	296	***Density(g/cm³):***	2.041		

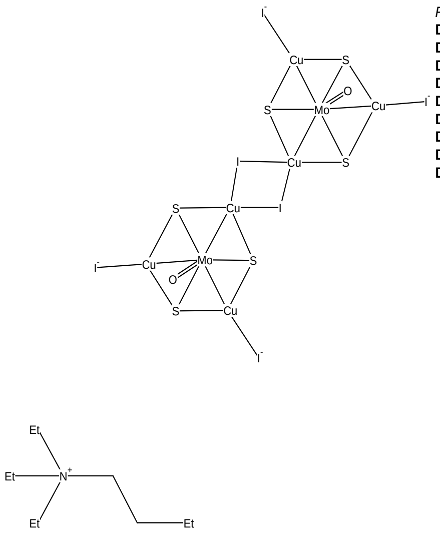

Parameters

Fragment 1

**DIST1 (D)**	3.421
**DIST2 (D)**	2.289
**DIST3 (D)**	2.293
**DIST4 (D)**	3.053
**DIST5 (D)**	2.498
**DIST6 (D)**	2.293
**DIST7 (D)**	2.289
**DIST8 (D)**	2.498
**DIST9 (D)**	3.053

Search: search2 (Fri Mar 27 10:46:00 2020): Hits 33-34

YOPPID

Reference: Yu-Jian Huang, Ying-Lin Song, Yang Chen, Hong-Xi Li, Yong Zhang, Jian-Ping Lang (2009) *Dalton Trans.* ,1411

Formula: $C_{54}\ H_{72}\ Cu_6\ I_2\ N_{12}\ S_8\ W_2,2(C_3\ H_7\ N_1\ O_1)$

Compound Name: tetrakis(μ_3-Sulfido)-bis(μ_2-1,3,5-tris(3,5-dimethylpyrazolyl)-2,4,6-trimethylbenzene)-tetrakis(μ_2-sulfido)-bis(μ_2-iodo)-hexa-copper(i)-di-tungsten dimethylformamide solvate

Space Group: P-1
Space Group No.: 2

Cell: ***(Å,°)*** ***a*** 12.650(1) ***b*** 12.943(3) ***c*** 15.041(3) α 111.39(1) β 95.08(2) γ 105.55(3)

R-Factor (%): 7.01 ***Temperature(K):*** 298 ***Density(g/cm^3):*** 1.763

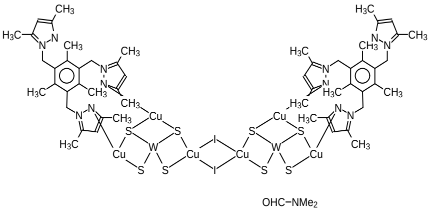

Parameters

Fragment 1

**DIST1 (D)**	3.298
**DIST2 (D)**	2.323
**DIST3 (D)**	2.287
**DIST4 (D)**	2.604
**DIST5 (D)**	2.693
**DIST6 (D)**	2.287
**DIST7 (D)**	2.323
**DIST8 (D)**	2.693
**DIST9 (D)**	2.604

ZIFFUR

Reference: T.S.Lobana, R.Sultana, R.J.Butcher, A.Castineiras, T.Akitsu, F.J.Fernandez, M.Cristina Vega (2013) *Eur.J.Inorg.Chem.* , 5161

Formula: $C_{12}\ H_{20}\ Cu_2\ I_2\ N_4\ S_8$

Compound Name: bis(μ_2-iodo)-tetrakis(1,3-thiazolidine-2-thione)-di-copper(i)

Space Group: P-1
Space Group No.: 2

Cell: ***(Å,°)*** ***a*** 7.597(0) ***b*** 9.226(0) ***c*** 9.609(0) α 96.51(0) β 109.54(0) γ 90.08(0)

R-Factor (%): 2.71 ***Temperature(K):*** 100 ***Density(g/cm^3):*** 2.261

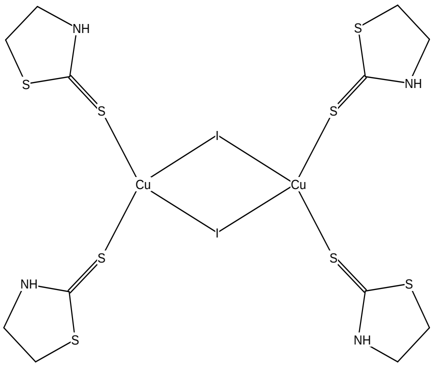

Parameters

Fragment 1

**DIST1 (D)**	3.567
**DIST2 (D)**	2.265
**DIST3 (D)**	2.270
**DIST4 (D)**	2.713
**DIST5 (D)**	2.757
**DIST6 (D)**	2.270
**DIST7 (D)**	2.265
**DIST8 (D)**	2.757
**DIST9 (D)**	2.713
